# Aerobic metabolic scope mapping of an invasive fish species with global warming

**DOI:** 10.1093/conphys/coad094

**Published:** 2023-11-28

**Authors:** Giovanni Quattrocchi, Emil Christensen, Matteo Sinerchia, Stefano Marras, Andrea Cucco, Paolo Domenici, Jane W Behrens

**Affiliations:** National Research Council, Institute for the study of the Anthropic Impact and Sustainability in the marine environment, Loc. Sa Mardini, 09170, Oristano, Italy; National Institute of Aquatic Resources, Technical University of Denmark, Kgs. Lyngby, Denmark; Institute of Biodiversity, Animal Health and Comparative Medicine, University of Glasgow, Glasgow, United Kingdom; National Research Council, Institute for the study of the Anthropic Impact and Sustainability in the marine environment, Loc. Sa Mardini, 09170, Oristano, Italy; National Research Council, Institute for the study of the Anthropic Impact and Sustainability in the marine environment, Loc. Sa Mardini, 09170, Oristano, Italy; National Research Council, Institute for the study of the Anthropic Impact and Sustainability in the marine environment, Loc. Sa Mardini, 09170, Oristano, Italy; National Research Council, Institute for the study of the Anthropic Impact and Sustainability in the marine environment, Loc. Sa Mardini, 09170, Oristano, Italy; National Research Council, Istituto di Biofisica, Pisa, Italy; National Institute of Aquatic Resources, Technical University of Denmark, Kgs. Lyngby, Denmark

**Keywords:** Baltic Sea, climate change, physiological model, round goby, thermal habitat suitability

## Abstract

Climate change will exacerbate the negative effects associated with the introduction of non-indigenous species in marine ecosystems. Predicting the spread of invasive species in relation to environmental warming is therefore a fundamental task in ecology and conservation.

The Baltic Sea is currently threatened by several local stressors and the highest increase in sea surface temperature of the world’s large marine ecosystems. These new thermal conditions can further favour the spreading of the invasive round goby (*Neogobius melanostomus*), a fish of Ponto-Caspian origin, currently well established in the southern and central parts of the Baltic Sea. This study aims to assess the thermal habitat suitability of the round goby in the Baltic Sea considering the past and future conditions. The study combines sightings records with known physiological models of aerobic performance and sea surface temperatures. Physiological models read these temperatures, at sighting times and locations, to determine their effects on the aerobic metabolic scope (AMS) of the fish, a measure of its energetic potential in relation to environmental conditions. The geographical mapping of the AMS was used to describe the changes in habitat suitability during the past 3 decades and for climatic predictions (until 2100) showing that the favourable thermal habitat in the Baltic Sea has increased during the past 32 years and will continue to do so in all the applied climate model predictions. Particularly, the predicted new thermal conditions do not cause any reduction in the AMS of round goby populations, while the wintertime cold ranges are likely expected to preserve substantial areas from invasion.

The results of this research can guide future monitoring programs increasing the chance to detect this invader in novel areas.

## Teaser

This paper investigates how predicted future thermal conditions (until 2100) in the Baltic Sea can favour the further spread of the invasive round goby. Sightings records, physiological models of aerobic performance and sea surface temperatures were used to assess the past and future thermal habitat suitability of the species.

## Introduction

Climate change is currently causing large-scale shifting in the geographical distributions of organisms. This is predominantly due to environmental warming, as species adapt their geographical range to maintain their optimal thermal niche (Pecl *et al.*, 2017). Previous works also suggest that redistribution of species due to climate change occurs markedly faster in the ocean than on land (Sorte *et al.*, 2010; Burrows *et al.*, 2011; Poloczanska *et al.*, 2013). Marine species redistribution creates novel ecological communities, which are not only significant for ecological balance, but also affect ecosystems services to humans through changed catch potential of important commercial fish species (Pecl *et al.*, 2017). Furthermore, climate change is expected to increase negative ecosystem effects of invasive species, altering ecosystem functioning, and changing the physical structure of habitats (Mooney and Hobbs, 2000; Hellmann *et al.*, 2008). Understanding and predicting invasive species range shifts with environmental warming is therefore a crucial topic in ecology and conservation (Ojaveer *et al.*, 2015; Giakoumi *et al.*, 2019).

Where biological processes are tied together with predicted environmental changes, mechanistic modelling provides strategic tools to evaluate species distributions. The adoption of mechanistic modelling to describe potential future distribution of species has the advantage of incorporating deterministic links between the functional traits of organisms, like fitness, and their environment (Kearney and Porter, 2009; Buckley *et al.*, 2010).

The aerobic metabolic scope (AMS) can measure the variation of the energetic potential of a fish to fuel all biological activities (movement, digestion, growth and reproduction) above those of standard metabolic rate and is often used as a proxy of fitness (Claireaux and Lefrançois, 2007; Chabot *et al.*, 2016). In ectotherms, AMS is inherently affected by the ambient temperature due to thermodynamic effects on biochemical processes and capacity limitations (Fry, 1947; Pörtner and Farrell, 2008; Schulte *et al.*, 2015). Therefore, it has been suggested as a good key trait to be used in mechanistic modelling of habitat suitability (Cucco *et al.*, 2012; Deutsch *et al.*, 2015; Marras *et al.*, 2015).

Coastal seas currently suffer from several environmental changes and threats, such as pollution and/or introduction of non-indigenous species that lead to rapid habitat changes and degradation (Reusch *et al.*, 2018). The Baltic Sea is an example of a sea that has been threatened by multiple stressors and recently defined as a ‘time machine for the future coastal ocean’ (Reusch *et al.*, 2018). It is a semi-enclosed brackish water basin located in northern Europe and characterized by a large drainage area with a significantly variable bottom topography. The considerable amounts of freshwater discharge determine a south–north-oriented salinity gradient, with near-freshwater conditions in correspondence of the Bothnian Bay and the inner part of the Gulf of Finland (Meier *et al.*, 2022). The water column is strongly stratified, and the sea temperature of the upper mixed layer is characterized by a seasonal cycle driven by solar radiation (Leppäranta and Myrberg, 2009). In winter, the water column is thermally mixed, and ice can cover up to 50% of the north-eastern part of the basin (HELCOM, 2007).

**Figure 1:**
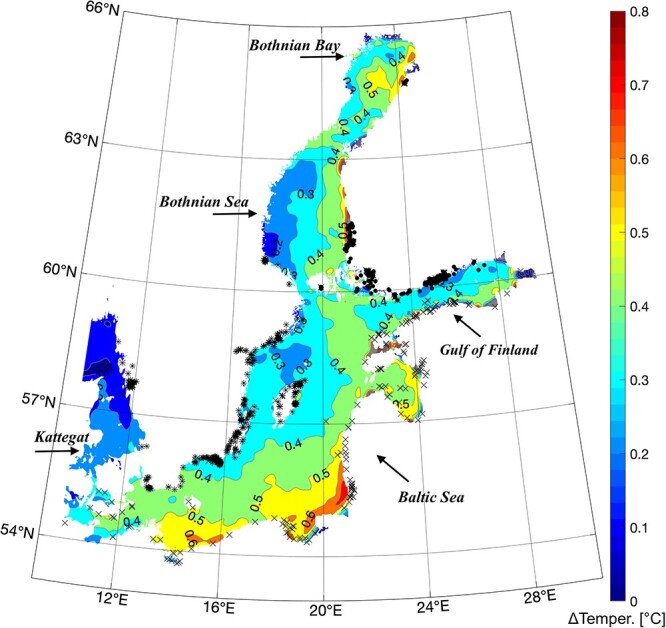
Sea surface temperature anomaly (°C) in the Baltic Sea region during the last decade (2010–19) and with respect to the last 30 years’ climatology (1990–2019), combined with round goby observations from Swedish (*), Finnish (•) and HELCOM (x) databases.

Significant ecosystem changes like environmental warming, acidification, nutrient pollution and deoxygenation have already been recorded in the Baltic Sea region (BACC II Team A, 2015; Reusch *et al.*, 2018). In fact, between 1982 and 2006 the sea surface temperature (SST) has increased more than any other large marine ecosystem (Belkin, 2009). Considering the most recent decade (2010–19), Fig. 1 displays a map of the summer SST anomaly with respect to the last 30 years’ climatology (1990–2019). Further SST increases are expected by the year 2100 based on climatic projections. The effects of warming will be compounded by additional stressors like eutrophication and pollution (Mooney and Hobbs, 2000; Stachowicz *et al.*, 2002; Hellmann *et al.*, 2008; Walther *et al.*, 2009) that will further perturb a currently changing ecosystem.

Temperature changes can affect the distribution of ectotherms in the Baltic Sea, where new thermal conditions have promoted a favourable habitat for non-indigenous species, such as the round goby (*Neogobius melanostomus; Pallas, 1814*), one of the most widely dispersed invasive fish in the world (Kornis *et al.*, 2012; Azour *et al.*, 2015; Puntila-Dodd *et al.*, 2021).

The round goby was introduced from its native range in the Ponto-Caspian region into temperate ecosystems throughout Europe and North America. This species has produced vast ecological impacts in colonized environments including the alteration of benthic invertebrate composition through predation (van Deurs *et al.*, 2021), predation on native fish eggs (Oesterwind *et al.*, 2017) and competition with native fish for food, shelter and spawning grounds (Karlson *et al.*, 2007; Ericsson *et al.*, 2021; Behrens *et al.*, 2022). Although the round goby exhibits a eurythermal AMS, its dispersal is likely limited to areas where temperatures allow for it to be competitive (Christensen *et al.*, 2021). Despite being introduced to the area ~30 years ago, round goby is still absent, aside from sporadic observations, from the north-eastern and coldest areas of the Baltic Sea (Puntila-Dodd *et al.*, 2021; ICES, 2022), yet its distribution is likely to expand beyond the current cold boundaries due to predicted climate change. However, the proportion of the currently uninvaded areas of the Baltic Sea that will become suitable thermal habitat for round goby, and how fast it can be expected to occur, remain uncertain.

In this study, the thermal habitat suitability of the round goby in the Baltic Sea considering the past and future conditions was assessed. This was achieved by combining occurrence data, physiological models of aerobic performance and oceanographic information. Given the round goby’s negative ecological effects in the Baltic region, such insights may aid the implementation of mitigation efforts and support management plans (Ojaveer *et al.*, 2015; Samson *et al.*, 2017). As a reference species for comparison, we also model future thermal habitat suitability, based on AMS, of the native Atlantic cod (*Gadus morhua*). Atlantic cod has an endemic population in the Baltic Sea where it coexists with round goby. Historically, Atlantic cod constitutes a commercially important resource for the area, but recently it has been considered in a critical state due to overfishing, increasingly unfavourable oxygen conditions, limited food availability and increasing infection loads with parasites (Eero *et al.*, 2015, 2023).

## Materials and Methods

### Physiological information

The physiological model from Christensen *et al.* (2021), based on the concept of AMS as a thermal energetic potential index (Fry, 1947; Claireaux and Lefrançois, 2007; Pörtner and Farrell, 2008), was used as a mechanistic predictor of thermal habitat suitability for the round goby. AMS (mgO_2_h^−1^) is calculated as the difference between the maximal metabolic rate (MMR) and the standard metabolic rate (SMR), which were directly measured in laboratory experiments as oxygen consumptions (MO_2_, mgO_2_h^−1^) at maximum level of activity (MMR) and at rest (SMR), under different temperatures, T (from 5 to 27°C). The variation in AMS as a function of temperature was modelled using the logistic regression in Equation 1:

(1) \begin{equation*} {\mathrm{AMS}}_{\mathrm{r}.\mathrm{goby}}=\frac{\mathrm{a}}{1+{\mathrm{e}}^{\left(-\mathrm{b}\cdotp \left(\mathrm{T}-\mathrm{c}\right)\right)}} \end{equation*}

where a = 13.645, b = 0.280 and c = 6.176. The model was based on 50-g fish.

A similar study for Atlantic cod (Tirsgaard *et al.*, 2015) calculated AMS (Eq.2) as the difference between MMR and SMR (Eq. 3), described by oxygen consumption under different temperatures T (from 2 to 20°C) and at different body mass (BM): 50, 200 and 450 g.

(2) \begin{equation*} {\mathrm{AMS}}_{\mathrm{a}.\mathrm{cod}}={\mathrm{MMR}}_{\mathrm{a}.\mathrm{cod}}-{\mathrm{SMR}}_{\mathrm{a}.\mathrm{cod}} \end{equation*}

where

(3) \begin{equation*} {\mathrm{MMR}}_{\mathrm{a}.\mathrm{cod}},{\mathrm{SMR}}_{\mathrm{a}.\mathrm{cod}}=\mathrm{a}\cdotp{\mathrm{e}}^{\left(\mathrm{b}\cdotp \mathrm{T}\right)}\cdotp{\mathrm{BM}}^{({\mathrm{a}}_1\cdotp{\mathrm{T}}^2+{\mathrm{b}}_1\cdotp \mathrm{T}\cdotp{\mathrm{c}}_1)} \end{equation*}

and the parameters are given in Table 1.

**Table 1:** SMR and MMR parameters for Atlantic cod, in Tirsgaard *et al.* (2015)

**Parameter**	**SMR**	**MMR**
a	0.0687	0.226
b	0.0596	0.0572
a1	−0.000892	−0.00074
b1	0.0211	0.0106
c1	0.788	0.870

The coefficients of Equations 1 and 2 were empirically derived from laboratory experiments performed on the two fish species with the differences reflecting their different ecophysiological response to temperature changes.

**Figure 2:**
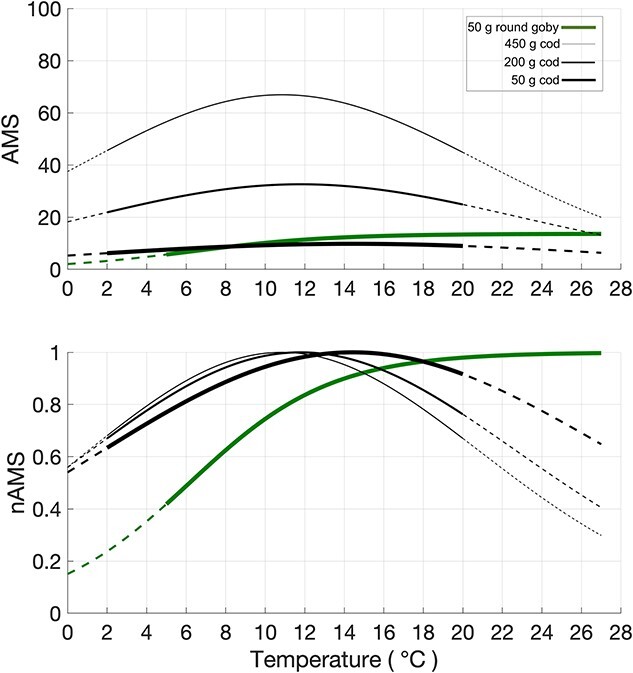
Top panel: AMS (mgO_2_h^−1^) of 50-g round goby (green line that is solid in the interval 5-27 °C Christensen *et al.*, 2021) and 50-, 200- and 450-g Atlantic cod (black lines that are solid in the interval 2-20 °C; Tirsgaard *et al.*, 2015); dotted lines refer to the extension of the curves beyond the temperature range used in the laboratory experiments. Bottom panel: AMS values normalized by maximum temperature (nAMS).

In Fig. 2, AMS values are normalized by the maximum experimental value to produce nAMS. The AMS (in the top panel) and nAMS (in the bottom panel) are displayed as a function of a wide range of sea temperatures. This temperatures range is based on those adopted by Christensen *et al.* (2021) and Tirsgaard *et al.* (2015) in their laboratory experiments. Using the established equations (Eq. 1 and Eq. 2), AMS and nAMS values were extrapolated from 0 to 27°C.

### Presence of round goby and thermal habitat assessment

The distribution of round goby in the Baltic Sea (Fig. 1) was derived by the HELCOM database (HELCOM, 2007) as well as a Finnish (FDB, 2021) and a Swedish (SDB, 2021) public database of observations presence; (see Supplementary Material, Fig. S1, S2, S3). HELCOM is a long-term set of data, aggregated by year, that was recorded during the years 1990, first observation of round goby in the Baltic Sea, to 2020. Finnish and Swedish databases describe the fish distribution along the coastal areas in space and exact date of observation. Observations have a temporal coverage of 12 years (2010–21) over all seasons, and they were mostly recorded during the temperate and warmer seasons.

SST observations, from 1990 to 2021, were used to obtain an assessment of the recent round goby thermal habitat, and they were obtained through the Climate Data Store of the Copernicus program (CDS, 2021). These data consist of a global area coverage of SSTs that are based on multi-sensors measurements from multiple polar-orbiting satellites and processed in the framework of the European Space Agency SST Climate Change Initiative. In correspondence of sea ice, SST values are posed to −0.33°C, the freezing temperature of seawater with salinity of 6 PSU (Stramska and Białogrodzka, 2015; Leppäranta and Myrberg, 2009, page 221). The temporal resolution of this dataset is diurnal with no gaps and the geographical resolution is defined into a regular grid of 0.05°, ~5 km. This dataset provides an accurate and validated estimate of the foundation temperature at sea (i.e. a temperature that is representative of a surface layer of 10 m depth) and its features make it suitable to assess climate spatial patterns, their variability, trends (CDS, 2021) and any potential ecological implications.

Considering the Finnish (FDB, 2021) and a Swedish (SDB, 2021) subset of sightings data, from 2010 to 2021, SSTs were estimated at the known time and geographical locations of round goby, via bilinear interpolation. Temperatures were obtained for 47% of the total sightings (equal to 2613), indeed, satellite observations are not available very close to the coasts (see Supplementary Material, Fig. S2).

According to the laboratory-derived relationship between nAMS and temperature (Fig. 2), the highest SSTs encountered during the warm season in the Baltic Sea, with a maximum of 19.5°C, do not negatively affect the thermal habitat suitably for round goby (i.e. nAMS > 0.97). Therefore, the present study focused on the effect of colder temperatures, which can instead drastically reduce the nAMS. The 25^th^ centile of the SSTs at sighting locations (7.7°C, corresponding to a nAMS of 0.6) was chosen as a threshold below which the nAMS of round goby is considered as suboptimal, due to the low sea temperatures that are typical of the winter season.

The nAMS values of round goby, used to map their thermal habitat, were assessed based upon this threshold, below which the habitat was considered unsuitable. A unitless suitability index, named ‘Aptness Index’, was designed to describe the interannual variation of the thermal habitat between the years 1990 and 2021. The ‘Aptness Index’ is defined as the ratio given by the area of the Baltic Sea that is suitable (i.e. above the threshold) and unsuitable (i.e. below the threshold) for round goby.

### Metabolic performance of round goby and Atlantic cod with climate changes

SST climate projections, from multiple global-scale climate models, implemented in the framework of the Intergovernmental Panel on Climate Change 5th assessment (IPCC5), were used to predict future nAMS in round goby and Atlantic cod from 2011 to 2100 in the Baltic Sea. The adoption of SST as a predictor for the spatial and temporal distribution of the nAMS of Atlantic cod may be limiting due to the known occurence of this species in deeper waters. However, SST is a proxy of the entire ocean water warming (DaNovaro *et al.*, 2017; CDS, 2021), being changes in temperature of deep waters linked to changes at the sea surface. Moreover, in the Baltic Sea, young adult cod, with size from 12 to 42 cm, may inhabit shallow waters primarily during cold and temperate seasons (McDonald *et al.* 1984). We used two complementary, freely available datasets, including high and low spatial resolution of SSTs fields. The dataset with high spatial resolution was used to assess with details the geographical variations of nAMS in round goby, while the low-resolution dataset was used to describe its interannual variability also in relation to the indigenous Atlantic cod.

SST projections of higher spatial resolution were derived from a public database (SMHI, 2022) of sea temperature estimated changes (EC) and averaged across seasons for three predetermined periods (2011–40, 2041–70 and 2071–2100), compared with a reference period 1976–2005. We added EC values to a reference period of satellite-sensed SST (1982–2005) to obtain future projections of sea temperatures and infer nAMS changes in the Baltic Sea. EC values were released by the Swedish Meteorological and Hydrological Institute and derived by an ensemble of three regional climate models, with spatial resolution of ~3.7 km, in the oceanographic part, and driven by 5 realizations of as many global climate models and two greenhouse gas concentration scenarios (RCP4.5 and RCP8.5; Kjellström *et al.*, 2018; Sørland *et al.*, 2018; Saraiva *et al.*, 2019; Gröger *et al.*, 2019).

SST projections of lower spatial resolution (see Supplementary Material, Fig. S4) were retrieved by the Climate Data Store (CDS, 2021) and consist of an ensemble of five climate models (EC-Earth, HadGEM-2-ES, MPI-ESM LR, CMCC-CMS and IPSL-CM5A-MR) and two scenarios (RCP4.5 and RCP8.5). It is a subset of the IPCC5—Coupled Model Intercomparison Project (CMIP5) computational effort that includes four models that were chosen by Meier *et al.* (2019) and Saraiva *et al.* (2019) to drive the high-resolution climatic regionalization in the Baltic Sea (SMHI, 2022).

SST fields are characterized by monthly frequency and a spatial resolution varying between models and roughly ~100 km.

## Results

**Figure 3:**
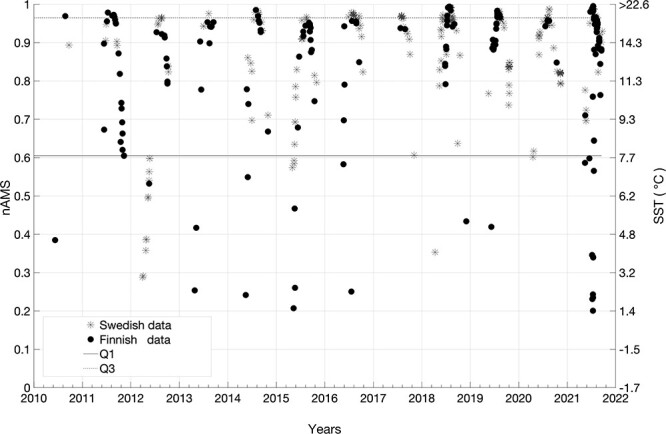
Estimated sea surface temperatures (SST at non-linear right y-axis) in correspondence of round goby observations. Round goby observations, from Swedish (*) and Finnish (•) databases, were recorded between the years 2010 and 2021. nAMS values, in the linear left y-axis, were computed according to the adopted physiological model (Eq. 1). Solid and dotted lines identify the nAMS interquartile range between the 25^th^ (Q1) and 75^th^ centile (Q3).

In Fig. 3, SSTs estimates (right y-axis) are displayed with the inferred nAMS values (left y-axis). The variance of the central 50% of the sample is given by the interquartile range and corresponds to the SSTs of the Baltic Sea temperate seasons. The limits of the nAMS interquartile range, the 75^th^ and the 25^th^ centiles (horizontal lines in Fig. 3), are equal to 0.97 and 0.6, corresponding to the SSTs of 19.0°C and 7.7°C, respectively. While the SSTs above the 75^th^ centile, typical of the warmer seasons, cannot negatively affect the nAMS of round goby (Fig. 2), temperatures below the 25^th^ centile, typical of the colder seasons, rapidly reduce the nAMS to low levels (in the range of 0.2–0.6).

The round goby is occasionally observed at times and locations where it seems to experience sea temperatures <5°C. However, there exist no laboratory experiments that could describe the metabolic performance of the round goby at such low temperatures, and nAMS values in Figures 2 and 3 were obtained according to the adopted physiological model (Eq. 1).

### Thermal habitat suitability in the past decades

**Figure 4:**
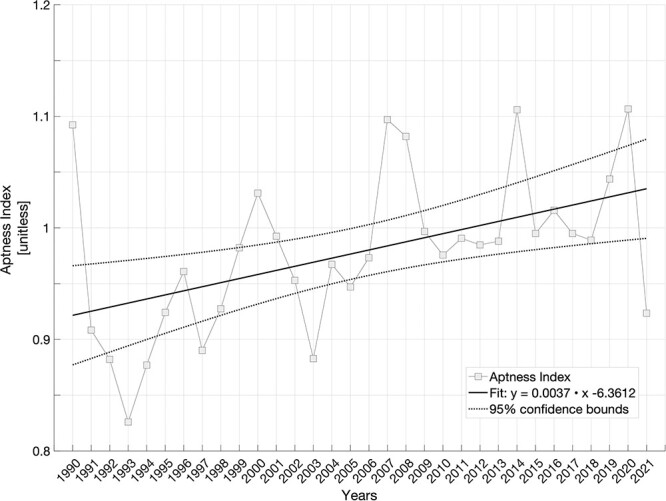
The ‘Aptness Index’ (unitless) computed as the ratio between suitable and unsuitable habitat area (%) of the round goby potential suitable thermal habitat since its first observation (1990) in the Baltic Sea (grey squares) and linear regression (solid line) with confidence interval (dotted lines). R^2^ = 0.23478.

Based on the 25^th^ centile threshold (nAMS = 0.6), a unitless ‘Aptness Index’ is used to describe the ratio between the yearly averages of total areas of suitable and unsuitable thermal habitat of the round goby in the Baltic Sea, between the years 1990 and 2021 (Fig. 4). Values >1 indicate that suitable areas, those characterized by favourable thermal conditions, are larger than unsuitable ones and vice versa.

The mean and the standard deviation of the ‘Aptness Index’ across the years 1990–2021 are 0.97 ± 0.07; between the years 1990 and 2006 are 0.94 ± 0.06 and the index is mostly <1; between the years 2007 and 2021 are 1.02 ± 0.05. This latter period includes the six highest global ocean heat contents between 2014 and 2019 (Cheng *et al.*, 2020) and a documented series of widespread marine heat waves in the neighbouring North Sea region and in the Baltic Sea (von Schuckmann *et al.*, 2021).

A linear fitting of the last 32 years’ ‘Aptness Index’ describes an increasing trend that is related to temperature increases in Europe, documented by regional climate modelling applications (e.g. Kjellström *et al.*, 2018).

**Figure 5:**
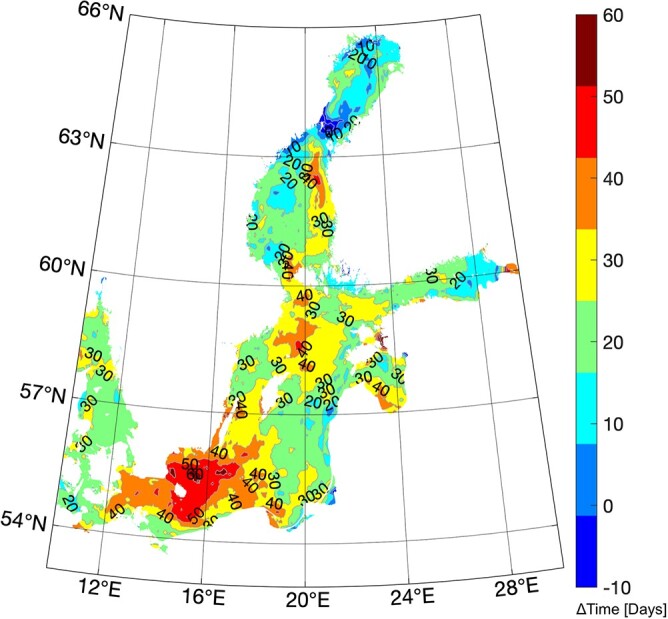
The difference (in days) between the number of days in 2020 and 2010 where the inferred nAMS values are found above the 25^th^ centile threshold in the Baltic Sea.

During the last decade, the geographical area of the suitable thermal habitat for the round goby population has increased steadily in the Baltic Sea (Fig. 5). The yearly number of days with favourable thermal conditions have increased everywhere, reaching maxima of 60 days (a 100% increase in 2020 compared to 2010) in the southernmost part of the Baltic Sea, 40 days (~66% increase in 2020 compared to 2010) in limited areas of its central part, and 20 days (~33% increase in 2020 compared to 2010) in the most northern part, the Bothnian Sea. Exceptions can be found at specific locations at the entrance of the Bothnian Bay.

**Figure 6:**
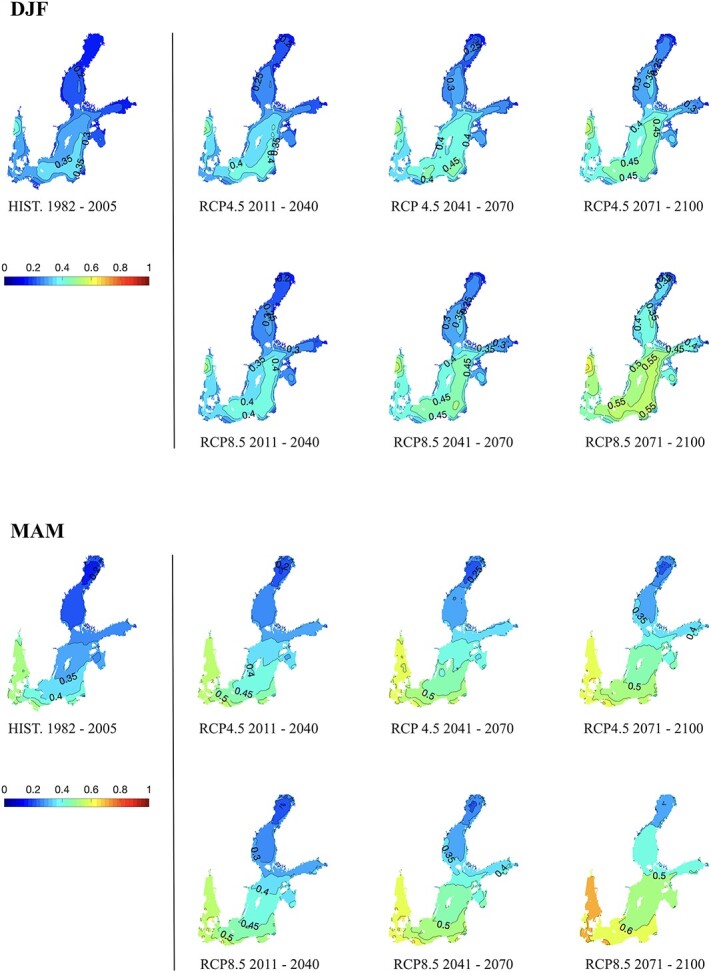
Maps of nAMS of round goby, per season. The acronyms DJF (December to February) and MAM (March to May) refer to the month’s names. Each panel shows the past (historical reference field: HIST. 1982–2005) and six 30-year climatological mean fields of future climate modelling projections (2011–41, 2041–70 and 2071–2100), for different greenhouse gas concentration scenario, RCP4.5 and RCP8.5.

**Figure 7:**
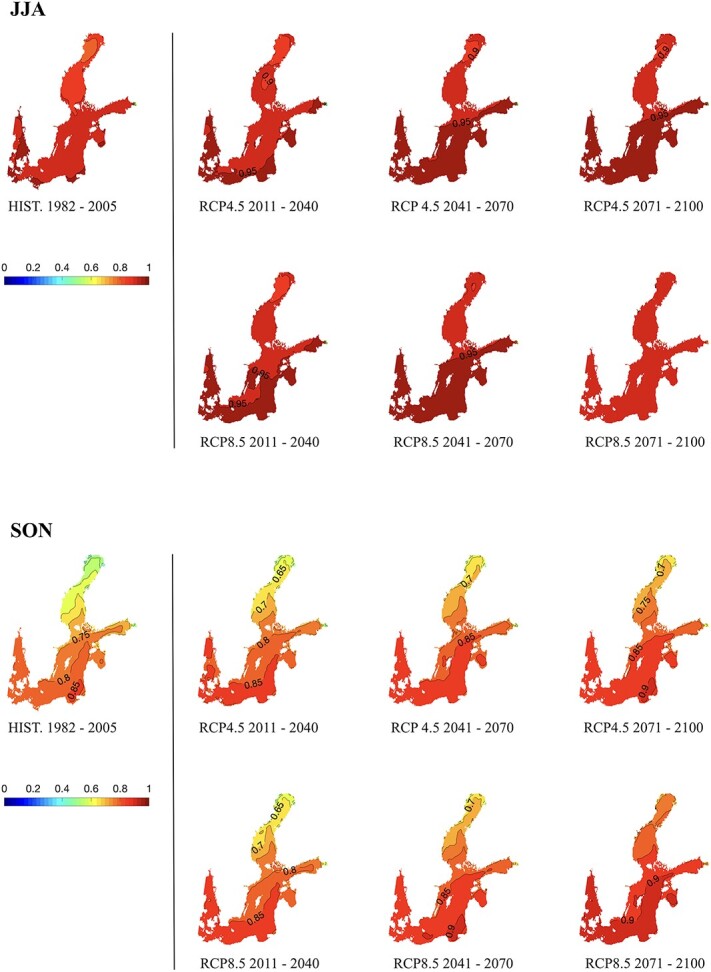
As in Figure 6 but for JJA (June–August) and SON (September–November).

**Figure 8:**
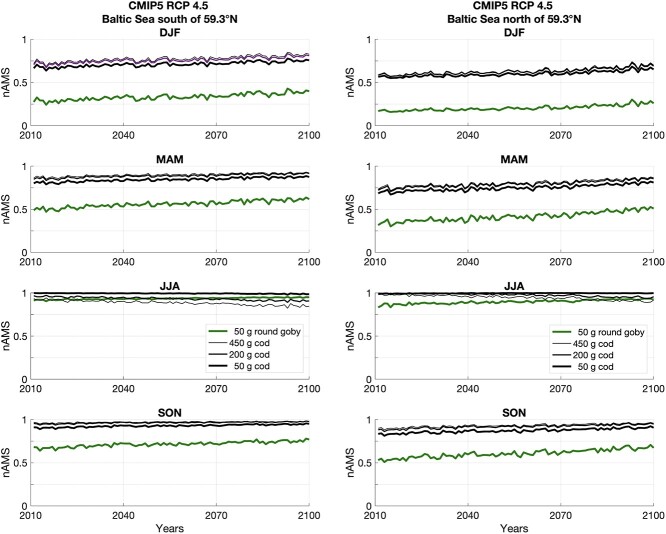
nAMS values are derived by SST information of a 5-model ensemble mean of climatic projections of the CMIP5, for greenhouse gas concentration scenario RCP 4.5 (IPCC 5th). They are yearly averaged and averaged in space, south of 59°N (left panel) and north of 59°N (right panel) in the Baltic Sea. nAMS of 50-g round goby (green lines) and 50-, 200- and 450-g Atlantic cod (black lines) are plotted per season (DJF, MAM, JJA, SON) from 2011 to 2100.

**Figure 9:**
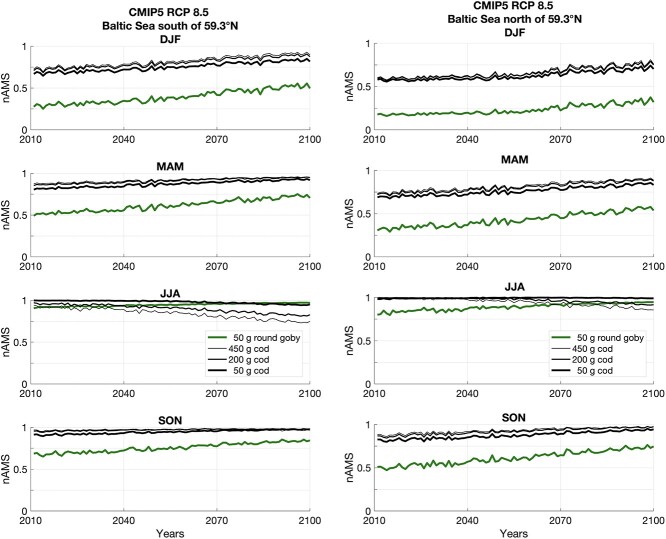
As in Figure 8 but for the greenhouse gas concentration scenario RCP 8.5 (IPCC 5^th^).

### Thermal habitat suitability and global warming

Considering the past climatology (Fig. 6 and 7, HIST. 1982–2005), a marked heterogeneity of the nAMS values is found both spatially and seasonally. During winter (December to February, DJF) the nAMS is generally <0.3 with diminishing values towards north and the innermost part of the Gulf of Finland. The spring months (March to May, MAM) display a steeper gradient along the south–north direction with maxima values equal to 0.5, and 0.6, but only west of the Danish Strait. The summer (June to August, JJA) is characterized by thermal conditions that determine the highest nAMS of the year, higher than 0.8 everywhere in the Baltic Sea. Thermal conditions in autumn (September to November, SON) yields nAMS values of 0.7–0.8 south of 60°N and a north–south-oriented gradient with minimum of 0.5 in correspondence of the Bothnian Bay. Winter and spring display nAMS ranging between 0.27 and 0.37, below the 25^th^ centile threshold. This results from unfavourable thermal conditions during the cold period that are not compatible with sufficiently high physiological performances of the round goby. By contrast, summer and autumn display high nAMS values.

Considering future projections for the RCP4.5 scenario (Fig. 6 and 7, RCP4.5 2011–40, 2041–70, 2071–2100), the main basin scale effect of the SST’s warming trend is represented by a pattern of high nAMS values that arises in the south-eastern part of the Baltic Sea (2011–40) and propagates toward the north as displayed by the maps of the succeeding 30-year climatological fields. This feature is found in all seasons except for the summer, when thermal conditions determine high values of nAMS in the past as in the future, according to model projections. Compared to RCP4.5, future projections of RCP8.5 scenario (Fig. 6 and 7, RCP8.5 2011–40, 2041–70, 2071–2100) show similar patterns with a steeper south–north gradient by the end of the century and a generalized spatial homogenization to high values of nAMS.

Considering the winter season in both RCP scenarios, by the end of the century, the whole area of the Baltic Sea extending south of roughly 59°N will display nAMS between 0.4 and 0.5, nearing the 25^th^ centile threshold. Nevertheless, the nAMS values in correspondence to the gulfs and bays will still display lower values (≤0.4).

The nAMS was also computed for the endemic Atlantic cod that, in the Baltic Sea, coexists with the round goby (Figs 8 and 9). Indeed, although adult cod are more likely to be found in deep waters in the Baltic Sea, young adult cod also inhabit shallow waters, primarily during winter, spring, and autumn, yet not summer (McDonald *et al.* 1984). Based on pan-Baltic catch data, Behrens *et al.* (2022) on the other hand have shown that round goby may be found in shallow waters from May to October and deeper than 50 m depth from January to April (the colder months).

According to local SST, predicted values of nAMS in Atlantic cod (black lines) will not decrease with global warming during winter, spring and autumn. However, unlike for round goby (green lines), sea warming will reduce the nAMS of 50-, 200- and 450-g cod during summer, mostly in the southern part of the Baltic Sea and if considering the RCP8.5 climate scenario.

## Discussion

Predicting success or failure of invasive species under specific environmental conditions is not trivial. By using oceanographic information and physiological models derived by laboratory experiments, this study shows that, in the Baltic Sea, the nAMS of the invasive round goby will not be compromised, based on both past and predicted future thermal conditions. The predicted temperatures (until 2100) will not even exceed the upper avoidance temperature (24.3 ± 0.7°C) determined by Christensen *et al.* (2021) and thus will not constitute a challenge for the presumed thermal safety margin (Sunday *et al.*, 2014) of round goby (8.1 and 8.5°C, at acclimation temperatures of 10 and 20°C).

However, sea temperatures in cold seasons reduce the nAMS values, likely limiting the round goby spreading. In fact, the 25^th^ centile threshold (0.6) of the nAMS values, which were inferred at times and locations where round goby presence was recorded (Fig. 3), occurs at temperatures (7.7°C) where physiological performance drops most rapidly (Fig. 2). This is reflected in a pronounced reduction of the Q_10_ at temperature <10°C, Q_10_ being an index of the factorial change in a biochemical rate with a 10°C temperature change (Christensen *et al.*, 2021). Decreases in nAMS with colder temperatures will inevitably impair the potential for physiological performance, such as locomotion and digestion (Claireaux and Lefrançois, 2007; Chabot *et al.*, 2016). This is supported by an observed reduction in food intake of round goby at temperatures <10°C, which, in turn, will limit the growth potential at these low temperatures (Lee and Johnson, 2005). They have demonstrated the temperature dependence of the laboratory-measured maximum daily specific consumption rate, explicitly related to growth.

More data on round goby sightings, especially recorded during the colder seasons, might improve the identification of the threshold. However, the aerobic scope curve and the round goby preferential temperature (21°C), both defined after the laboratory experiments (Christensen *et al.*, 2021), suggest that the species should avoid very low sea temperatures that are typical of the colder seasons in the Baltic Sea. Furthermore, according to Lee and Johnson (2005), the consumption rate starts to significantly increase in the range of 5–10°C, while the nAMS threshold (0.6) in the present study corresponds to 7.7°C.

### nAMS of round goby in the past and future predictions

The ‘Aptness Index’ (the ratio between suitable and unsuitable areas of the Baltic Sea, based on nAMS) showed that the geographical area for a favourable thermal habitat for round goby in the Baltic Sea has increased throughout the past 32 years (Fig. 4). More specifically, in the northernmost and coldest regions, during the last decade, the number of days with favourable thermal conditions has increased by 20 additional days (Fig. 5). The first observations of round goby in this area of the Baltic Sea were recorded between 2010 and 2014, and although the presence of round goby has been recorded in the field at temperatures as low as 3–4.5°C (Behrens *et al.*, 2022), the fish seem reluctant to occupy cold waters, and there have been only few additional observations in this northernmost area in the period between 2014 and 2022 (Kotta *et al.*, 2016; ICES, 2022). Introductions mainly occur to ports via shipping (Kotta *et al.*, 2016; Holmes *et al.*, 2019), followed by secondary dispersal (Azour *et al.*, 2015; Christoffersen *et al.*, 2019). Relatively low shipping intensity in these northern areas (Kotta *et al.*, 2016; Holmes *et al.*, 2019) combined with the assumed poor performance at, and avoidance of, low temperatures (Christensen *et al.*, 2021), might explain the relatively low success of round goby in these northern regions. Yet, when living at these high latitudes, the cold (and food-poor) winter is to some extent unescapable. Winter dormancy is used by many animals to survive this challenging period of the year, and the stopping of, or greatly reducing, activity and feeding may in some fish species be supplemented by metabolic rate depression, an active downregulation of resting cellular energy turnover and thus SMR. This has e.g. been shown in winter-dormant cunner (*Tautogolabrus adspersus*) (Gerber *et al.*, 2022). Whether round goby is capable of this remains to be elucidated.

Notably, the results obtained in the present study show that, during the past decades, the climatic conditions have favoured the round goby population inhabiting the Baltic Sea and the rising temperatures will not mitigate their performance and dispersal potential in the future, until 2100. Yet, some seasonal differences persist. Indeed, considering the nAMS in the past climatology (HIST. 1982–2005 maps in Fig. 6 and 7), warmer seasons may represent a temporal window during which the round goby can spread and explore new favourable habitats, enabling further range expansion.

nAMS mapping with global warming (i.e. RCP4.5 and RCP8.5 scenarios in Fig. 6 and 7) also showed that the natural variability of the seasonal cycle clearly represents a limitation for the increasing trend of the ‘Aptness Index’. Indeed, while the predicted nAMS fields, from early summer to autumn, indicate that the settlement of round goby can be favoured, the winter and spring fields of nAMS display large areas with unfavourable conditions (<0.5) for potential settlements, especially the Gulf of Finland and the Bothnian Sea and Bay. The persistence of the ice cover during the colder seasons, which can modify the albedo and the air-sea fluxes, is a primary factor affecting the local thermal conditions (Dutheil *et al.*, 2022) and the related nAMS patterns.

In summary, despite the increasing trend of the SST in future climate projections, the nAMS values obtained for winter and spring seasons may still prevent rapid dispersal and settlement of round goby in the most northerly region for both scenarios, RCP4.5 and RCP8.5. By contrast, the winter and spring seasons in the southern region are becoming more favourable for this fish that seems to seek relatively warm waters (Christensen *et al.*, 2021; Behrens *et al.*, 2022). Warmer water temperatures also allow for a longer breeding season, in addition to faster embryo development and reproductive rate, as known for the sand goby *Pomatoschistus minutus* (Pallas, 1770; Kvarnemo, 1994). Warm summers on the contrary may limit the nAMS of cold-adapted species, such as cod, in this region, and this is likely most critical for embryos and breeding adult fishes (Dahlke *et al.*, 2020).

### nAMS of Atlantic cod in future predictions

The Eastern Baltic cod stock is in distress and potentially on the verge of collapse (Eero *et al.*, 2015, 2023). This collapse may be caused by an interplay of multiple drivers including unfavourable climatic conditions, reduced quantity and quality of food, and an increasing grey seal population, which besides predation transmit parasites to the cod (Casini *et al.*, 2016; Sokolova *et al.*, 2018; Neuenfeldt *et al.*, 2019; Ryberg *et al.*, 2020).

Considering the worse IPCC climate scenario (RCP8.5; Fig. 9), the present results show that irrespective of size (50, 200 and 450 g) the nAMS of cod during summer decreases because of the expected warming. This is more evident for a 200- and 450-g cod. Besides the potential negative effect of nAMS on the capacity for physiological performance, cod also experience food limitations in this region (Casini *et al.*, 2016; Neuenfeldt *et al.*, 2019), which is why colder waters are preferred to lower metabolic rates and increase food conversion efficiency (Björnsson, 2019), thus leaving cod in ‘double trouble’ in a future warmer world. In addition, because temperature performance curves in general decrease more rapidly above than below the optimum temperature, limitations in physiological performance above the optimum temperature suggestively have more severe consequences than limitations below the optimum, due to increase in basal maintenance energy demand with increasing temperatures (Martin and Huey, 2008). Thus, although cod experience more favourable thermal habitat during some parts of the year, they are facing a decrease in nAMS in the warm part of the year, which round goby are not. This is more evident for a 450-g cod.

Notably, global warming is not only expected to increase the average environmental temperature, but also to increase the frequency and amplitude of heat waves (e.g. von Schuckmann *et al.*, 2021), which will only emphasize potential negative consequences for cod that the round goby will likely not be challenged by.

### Limitation of the nAMS approach

Deutsch *et al.* (2015) suggest that constraints on aerobic scope are the main limiting factors governing range limit for diverse marine ectotherms. According to them, a metabolic index (based on aerobic scope) can be used as a metric to predict future distribution, in line with Cucco *et al.* (2012) and Marras *et al.* (2015). Similarly, the results of Payne *et al.* (2016) support the idea that aerobic scope is an important factor limiting the distribution of marine ectotherms.

Nevertheless, the extent to which the relationship between temperature and aerobic scope affects the distribution and climate change sensitivity of ectotherms is currently debated (Clark *et al.*, 2013; Farrell, 2013; Pörtner and Giomi, 2013; Jutfelt *et al.*, 2018). Some authors suggest that the relationship between temperature and aerobic scope is not always a good predictor of temperature performance (Clark *et al.*, 2013; Norin *et al.*, 2014; Jutfelt *et al.*, 2018), whereas others argue that this relationship may be a good predictor of performance at the extremes but not at temperature optima (Verberk *et al.*, 2016; MacMillan, 2019; Khaliq *et al.*, 2023).

In addition, while many species show a decline in metabolic scope at higher temperatures (e.g. Reynolds and Casterlin, 1979; Habary *et al.*, 2017), the metabolic scope can in some species keep increasing above the preferred temperatures and optimal growth temperature levels (e.g. Norin *et al.*, 2014). Furthermore, based on Lee and Johnson (2005), who measured thermal growth performance, there is seemingly a close correlation between thermal performance of metabolic scope and thermal growth performance, yet as evident of Christensen *et al.* (2021), round goby does prefer lower temperatures than the highest temperature of high aerobic metabolic scope (this is interpreted as a thermal safety margin, (cf. Sunday *et al.*, 2014). Given all the above considerations, our results are therefore to be taken with caution when extrapolating the prediction of actual animal distributions and are intended to provide mainly a direct link to the future aerobic scope conditions of round gobies in the Baltic Sea through time and space. Because of the limitations on using aerobic scope for predicting ectotherm distribution, future works (as suggested by Wolfe *et al.*, 2020), should aim at incorporating various physiological performance metrics (e.g. swimming performance), temperature exposure and ecological data (e.g. species interactions, habitat type), to improve models of prediction of future distribution patterns.

### nAMS for management and conservation

The increasing importance of round goby into the Baltic trophic chain (Karlson *et al.*, 2007; Ojaveer *et al.*, 2015; Oesterwind *et al.*, 2017; Ericsson *et al.*, 2021; van Deurs *et al.*, 2021; Behrens *et al.*, 2022) makes the management of this invasive species a priority for biological conservation. In this context, ecological modelling offers numerous approaches and techniques for predicting future spatial distribution of invasive species and suggesting effective management strategies. Ecological models should—to the extent this information is available—aim at incorporating the thermal safety margins of the species at different life stages to enhance the reliability of the projected changes. Thermal habitat suitability techniques (e.g. nAMS mapping) could potentially benefit from integrating additional modelling approaches that have been recently used to describe the introduction processes, the dispersal and the distribution of suitable habitats for the round goby in Baltic and Norwegian coastal waters (Kotta *et al.*, 2016; Samson *et al.*, 2017; Holmes *et al.*, 2019; Forsgren and Hanssen, 2022).

The nAMS, as a predictor of thermal habitat suitability in future climate scenarios, can support the implementation of monitoring programs for this invader, for instance, by identifying the most likely areas for invasion, also outside the Baltic Sea. Besides, the application of the ‘Aptness Index’ can be used to assess, monitor or predict the impact of the invader, considering the extent of suitable or unsuitable areas.

Management of the Baltic ecosystem and fisheries stock should consider that predicted future increase in water temperature will inevitably push some species out of their optimal thermal window, as in the case of cod, while favouring some invasive species, as for example round goby. This might have a profound effect on the Baltic ecosystem’s future functioning, in terms of increased competition with invasive species, altered stock size structure and trophic interactions and species geographical displacement (Pecl *et al.*, 2017). Repercussions on the social-economic dimensions, in terms of reduced catches of species with reduced thermal adaptation flexibility and introduction of new fisheries for invasive species might hence be expected.

## Supplementary Material

Web_Material_coad094

## Data Availability

The data underlying this article are available at: https://fyndkartor.artfakta.se/https://www.smhi.se/en/climate/future-climate/about-the-analysishttps://vieraslajit.fi/havainnot?taxonId=MX.53000https://cds.climate.copernicus.eu/
