# Supplementary material for: Aerobic metabolic scope mapping of an invasive fish species with global warming
Source: Conserv Physiol. 2023 Nov 28;11(1):coad094. doi: 10.1093/conphys/coad094 (PMC10904007; doi:10.1093/conphys/coad094)
Supplement: Web_Material_coad094 [file web_material_coad094.zip › supporting_information.pdf]

Supplementary Material

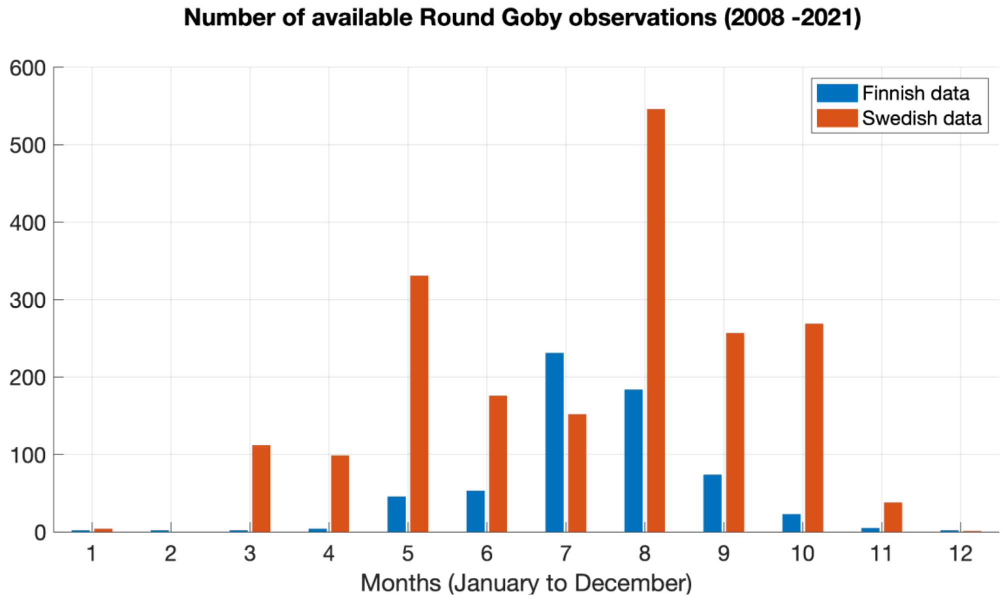

Figure S01. Monthly distribution of round goby observations between the years 2008 and 2021, derived by a Finnish and a Swedish public database (see references: FDB and SDB). Observations were recorded from January to December, the majority of the observations were recorded from March to October.

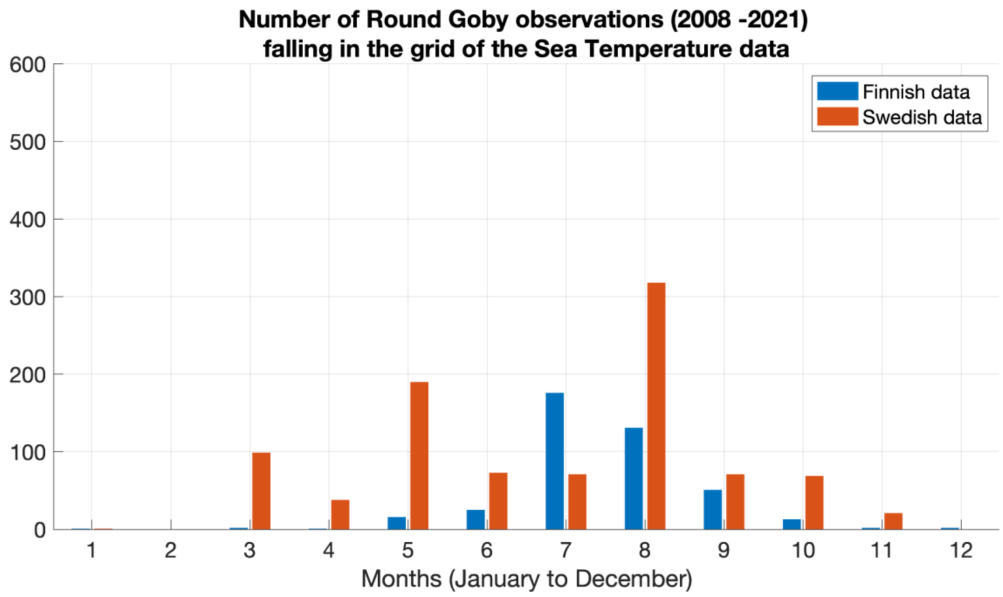

Figure S02. The percentage of round goby observations falling within the gridded sea surface temperature fields is 33 and 55 % of the total Finnish and Swedish dataset, respectively.

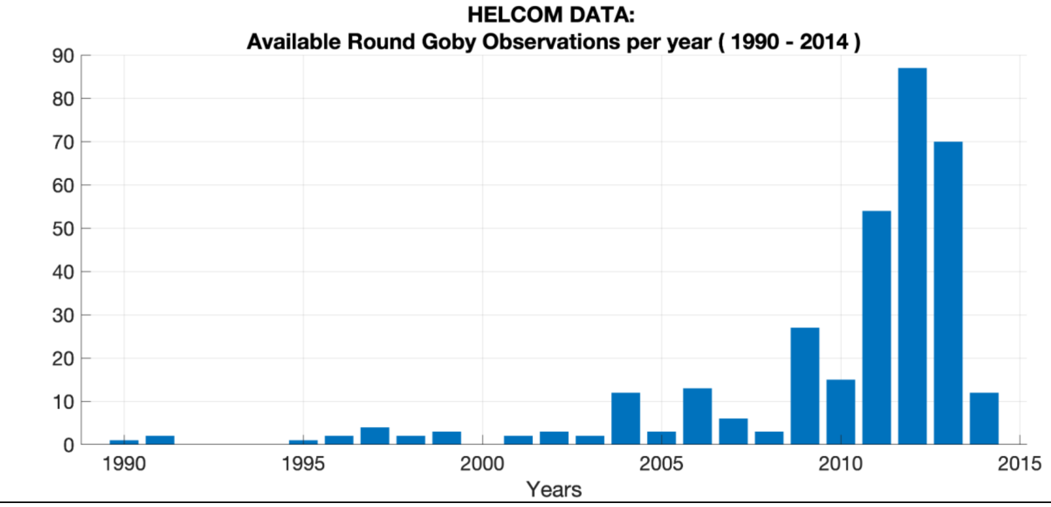

Figure S03. Yearly distribution of round goby observations derived by HELCOM dataset. Most observations are collected from 2004 to 2014.

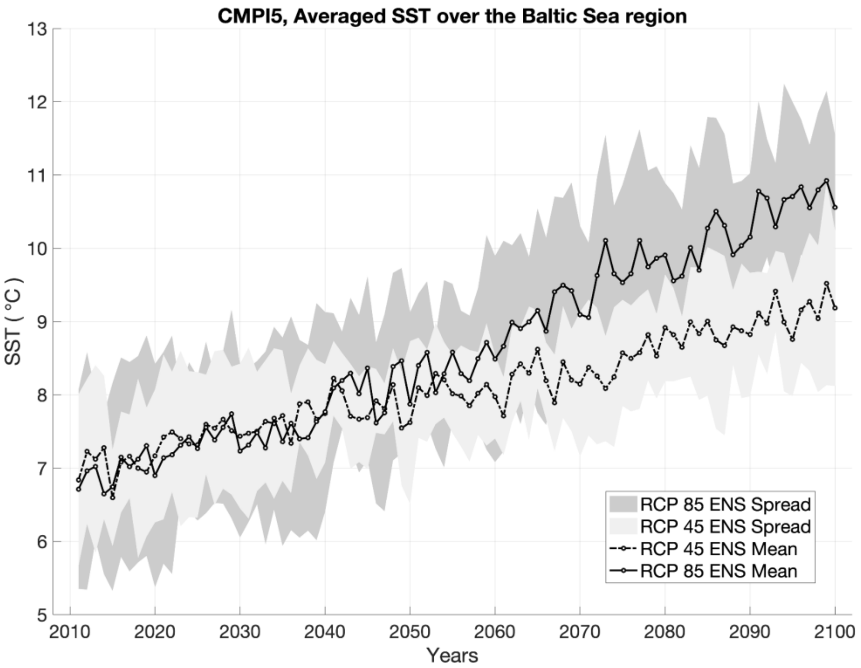

Figure S04. Available SST projections of the Baltic Sea from the Intergovernmental Panel on Climate Change (IPCC), 5th assessment, *Coupled Model Intercomparison Project* 5 and the variance of 5 models used in Figures 7.
